# Predicting stroke severity with a 3-min recording from the Muse portable EEG system for rapid diagnosis of stroke

**DOI:** 10.1038/s41598-020-75379-w

**Published:** 2020-10-28

**Authors:** Cassandra M. Wilkinson, Jennifer I. Burrell, Jonathan W. P. Kuziek, Sibi Thirunavukkarasu, Brian H. Buck, Kyle E. Mathewson

**Affiliations:** 1 Department of Psychology, P-455 Biological Sciences, Faculty of Science, University of Alberta, Edmonton, AB T6G 2E9 Canada; 2 Neuroscience and Mental Health Institute, Faculty of Medicine and Dentistry, University of Alberta, Edmonton, Canada; 3 Division of Neurology, Faculty of Medicine and Dentistry, University of Alberta, Edmonton, AB T6G 2R3 Canada

**Keywords:** Neuroscience, Health care, Medical research, Neurology

## Abstract

In this study, we demonstrated the use of low-cost portable electroencephalography (EEG) as a method for prehospital stroke diagnosis. We used a portable EEG system to record data from 25 participants, 16 had acute ischemic stroke events, and compared the results to age-matched controls that included stroke mimics. Delta/alpha ratio (DAR), (delta + theta)/(alpha + beta) ratio (DBATR) and pairwise-derived Brain Symmetry Index (pdBSI) were investigated, as well as head movement using the on-board accelerometer and gyroscope. We then used machine learning to distinguish between different subgroups. DAR and DBATR increased in ischemic stroke patients with increasing stroke severity (p = 0.0021, partial η^2^ = 0.293; p = 0.01, partial η^2^ = 0.234). Also, pdBSI decreased in low frequencies and increased in high frequencies in patients who had a stroke (p = 0.036, partial η^2^ = 0.177). Using classification trees, we were able to distinguish moderate to severe stroke patients and from minor stroke and controls, with a 63% sensitivity, 86% specificity and accuracy of 76%. There are significant differences in DAR, DBATR, and pdBSI between patients with ischemic stroke when compared to controls, and these effects scale with severity. We have shown the utility of a low-cost portable EEG system to aid in patient triage and diagnosis as an early detection tool.

## Introduction

Stroke is a devastating disease and the leading cause of disability in Canada^1^. For patients with ischemic stroke, early reperfusion with either thrombolysis or endovascular devices is the most effective means of saving brain tissue and improving recovery. Stroke diagnosis begins in the prehospital setting. Emergency medical services (EMS) crews examine patients with stroke symptoms using standardized scales that are designed to separate stroke from other conditions that mimic strokes before making transport decisions regarding the destination hospital^2^. Ensuring an accurate prehospital stroke diagnosis is essential to ensure that stroke patients are efficiently transported to hospitals that offer emergent reperfusion therapies while avoiding unnecessary transport of stroke mimics. Strokes scales assign points to clinical deficits based on an abbreviated version of the neurological exam. These prehospital stroke scales miss up to 30% of acute strokes^3^ and often miss stroke from large vessel occlusions (LVO) that benefit from endovascular thrombectomy^4^. There remains a pressing need for rapid, cost-effective technologies to improve the prehospital diagnosis of stroke^5^.

Electroencephalography (EEG), or recording the brain’s electrical activity, has been explored as a diagnostic and prognostic tool in stroke^6,7^. When cerebral blood flow (CBF) drops during ischemia, abnormalities such as decreases in fast frequencies are observed^8^. Abnormalities, including slowing (increased delta to alpha ratio, DAR or increased (delta + theta)/(alpha + beta) ratio, DTABR) and changes in brain symmetry, are well established in the acute phase of stroke^9,10^. The power spectrum begins to return to normal in the first 3 months after stroke, but quantitative EEG (qEEG) can still show abnormalities, such as impaired symmetry, that persist past the point of good motor recovery^11^. Past studies have shown that qEEG indices such as DAR can be used for both diagnosis of ischemic stroke^6,8,12,13^ and prediction of clinical outcomes^7,14–18^.

The majority of strokes are unilateral, and thus deficits (e.g., motor impairments) are lateralized as well^19^. Therefore, brain symmetry is another qEEG measure that has been used to distinguish stroke from control populations^7,8,12–14,18^. The brain symmetry index was first used during carotid surgery as a reliable method to detect early brain ischemia^20^. Modified from the brain symmetry index, the pairwise-derived brain symmetry index (pdBSI) calculates asymmetry by calculating the absolute value of the difference in power at each pair of electrodes^14^. Therefore, a larger pdBSI represents more asymmetry between the hemispheres. Higher pdBSI is often associated with worse outcome^7,10,21^. However, it has also been shown that pdBSI may vary based on stroke location, with cortical strokes having more symmetrical activity compared to subcortical strokes^12^.

These qEEG measures of post-stroke brain activity have also been found in animal models. These preclinical studies both validate findings seen clinically and provide continuous data over a longer period, giving insight into the electroencephalographic changes over the course of stroke recovery. After a transient middle cerebral artery occlusion (MCAO) in rats, DAR increases in the acute phase and begins to recover in the chronic phase^22^. Behavioural deficits correlated strongly with DAR in this rodent model. Additionally, although DAR recovers spontaneously after stroke, DAR recovers more efficiently in animals that underwent reperfusion after MCAO compared to those given a permanent MCAO^23^. In this large stroke model, a decrease in brain symmetry is observed, primarily in the regions affected by the stroke.

Despite successes finding predictive quantitative EEG measures, barriers exist that currently prevent EEG from being routinely used clinically as a diagnostic or monitoring device. Commonly, the set-up and recording of typical EEG devices can take valuable time, with many set-ups in past studies using nineteen or more electrodes^16^. In an acute and time-sensitive setting, such as an ambulance or an emergency room, the routine set-up of these systems would not be feasible. Further, access to both EEG devices and expertise can be limited. Demand exists for an EEG device that is cost-effective, simple to set-up, and easy to interpret. Advances in technology have led to consumer wireless EEG systems that meet these requirements. The Muse by InteraXon Inc. is one type of wearable consumer EEG system that is commercially available and has been compared to medical EEG devices^24^. Ratti et al. found the Muse required significantly less time to set up than the medical system, although this was accompanied with increased test–retest variability, lower data quality, and more artifacts due to eye blinks and muscle movement^25^. Despite these limitations, wearable EEG has been successful in diagnostic feasibility for other disorders, such as epilepsy^26^. Recently a pilot study was published that suggested stroke patients could be distinguished from healthy controls using the Muse headband due to significantly increased brain asymmetry in stroke patients^27^. However, this study used healthy volunteers as controls rather than stroke mimics, and it is possible that stroke mimics also present with EEG abnormalities. Further, only brain symmetry was assessed, and it is unclear if additional qEEG measures, such as slowing, would improve diagnostic accuracy. Finally, they did not determine the sensitivity of brain asymmetry as a measure to predict stroke occurrence, so further research is needed. If the data acquired using the Muse has enough sensitivity to distinguish stroke patients from controls, this inexpensive and portable device could be easily implemented in ambulances for improved diagnosis and triage to determine the most appropriate hospital (e.g., primary stroke centre).

The main aim of this study was to examine the use of a low-cost, portable EEG system in a subacute stroke population to distinguish ischemic stroke patients from a control group that included patients who presented with suspected stroke (i.e., “stroke mimics”). Further, we determined if EEG data can be used to detect differences in stroke severity. We used random forest classification trees to determine the most predictive combination of EEG and other physiological parameters, then calculated the sensitivity and specificity of these models in predicting stroke presence and stroke severity.

## Methods

### Ethics approval and consent

Written informed consent was obtained from participants or their legal guardians if they could not consent prior to participating. All methods were performed in accordance with the guidelines and regulations of the University of Alberta. The Human Research Ethics Office, University of Alberta, Canada, approved ethics for this study.

### Participants

Twenty-one patients presenting with stroke deficits were prospectively recruited from the stroke unit at the University of Alberta Hospital (Edmonton, Alberta). The diagnosis of ischemic stroke and stroke severity was confirmed by a neurologist blind to the EEG results, based on a review of the clinical information and neuroimaging that included multimodal CT and diffusion-weighted MRI. Stroke severity was graded based on the National Institutes of Health stroke scale (NIHSS) and size of infarct. Of the 21 patients, five had negative diffusion-weighted MRI and were included in the control group. The patients’ family members were recruited as an age-matched control, bringing our total sample to 25 participants (16 stroke patients and nine controls). Due to connectivity issues, two additional sessions were excluded from the analysis. Demographic information for each participant is available in Table 1. Participants were between the ages of 19 and 91 (mean age of 66). The mean recording time was 3.85 days post-stroke onset, and the average NIHSS was 7.7. Table 1 provides a summary of demographic information sorted by the severity of the stroke event.

**Table 1:** Participant demographics and characteristics and summary by severity grouping.

Subject number	Stroke or control	Severity	Stroke location	Lateralization	Time from stroke onset	NIHSS	Gender	Age
1	Control	Control	–	–	–	–	M	91
2	Stroke	Small	Cortical	Left	6 days	0	M	87
3	Stroke	Moderate	Cortical	Left	7 days	8	F	61
4	Stroke	Moderate	Cortical	Left	1 day	12	M	65
5	Stroke	Moderate	Subcortical	Left	3 days	16	F	83
6	Stroke	Small	Subcortical	Right	3 days	0	F	19
7	Stroke	Moderate	Cortical	Right	2 days	1	F	71
8	Stroke	Moderate	Cortical	Left	4 days	14	M	71
9	Stroke	Small	Cortical	Left	1 day	8	F	86
10	Stroke	Moderate	Cortical	Left	0 days	10	M	85
11	Stroke	Moderate	Cortical	Left	8 days	15	M	37
12	Stroke	Large	Subcortical	Right	16 days	10	M	87
13	Control	Control	–	–	–	–	M	66
14	Stroke	Large	Cortical	Left	2 days	14	F	53
15	Control	Control	–	–	–	–	M	53
16	Stroke	Small	Cortical	Left	3 days	1	M	66
17	Control	Control	–	–	–	–	F	64
18	Control	Control	–	–	–	–	F	81
19	Stroke	Moderate	Cortical	Left	2 days	2	M	75
20	Control	Control	–	–	–	–	M	56
21	Stroke	Moderate	Cortical	Left	0 days	10	M	87
22	Control	Control	–	–	–	–	M	59
23	Control	Control	–	–	–	–	F	48
24	Stroke	Large	Cortical	Right	6 days	10	F	72
25	Control	Control	–	–	–	–	M	29
**Summary data**								
	–	Control (9)		N/A	N/A	N/A	67% Male	60.8
	–	Small (5)		75% Left	3.25 days	2.25	33% Male	64.5
	–	Moderate (9)		89% Left	3 days	9.78	67% Male	70.6
	–	Large (3)		67% Left	8 days	11.33	50% Male	70.7

### Procedure

Each experiment began with an explanation of the procedure to the participant or their guardian, and then receiving informed consent. The participant’s forehead and earlobes were cleaned with NuPrep, an exfoliating gel, then cleaned with alcohol swabs to ensure a stable connection. The Muse (InteraXon Inc., Toronto, ON) was cleaned with alcohol swabs before and after each recording. EEG recordings consisted of two sessions, three minutes in an eyes-open resting state and another three minutes in an eyes-closed resting state. Eyes-open resting-state consisted of the patient concentrating at a fixation cross centred on a 13″ MacBook Air Laptop running macOS High Sierra version 10.13.3. Recordings were taken with a Muse headband that had its hard-plastic exterior removed and replaced with a soft headband, secured with an adjustable elastic cord strap, for improved electrode connection. The Muse was further modified as the ear electrodes were replaced with clip-on electrodes to ensure a better connection. The headband sat roughly 2.5 cm above the eyebrows. All electrodes were silver chloride coated and connected with electrolyte gel to further increase connectivity.

### EEG analysis

The Muse has a 256 Hz sampling frequency. The Muse consists of seven electrodes, four electrodes located at A7, A8, T9 and T10, a ground electrode located at Fpz, and two reference electrodes to the left and right of the ground. These electrodes roughly correspond to the international 10–20 electrode system. The Muse has an on-board digital signal processing module that performs noise filtering. Data was recorded using MuseLSL^28^. Data was then transmitted at 10 Hz via a Silicon Labs Bluetooth Module (Model: BLED112), for further analysis in MATLAB.

Oscillations in the data were estimated using a wavelet transform with a wavenumber of 15. Power was calculated for frequencies between 0.5 and 31 in steps of 0.1. Wavelet code was used from the BOSC (Better OSCillation detection) toolbox^29^. Only data from the eyes closed portion of the recording was used for further analysis, as some patients were unable to keep their eyes open for the three-minute recording window. Only TP9 and TP10 electrodes were used for qEEG measures, as some patients had connectivity issues with the frontal electrodes. Spectral power was calculated at each frequency at each time point for each participant. The entire three-minutes of processed EEG data was then averaged across time to create a single power spectrum at each electrode for each participant. Then the global asymmetry was calculated using homologous electrode pairs in the 1–30 Hz range. We used the calculation from Sheorajpanday et al. 2011, which defined pdBSI as:

1 $$pdBSI=\sum_{j=1}^{M}\sum_{i=1}^{N}\left|\left.\frac{{(R}_{ij}-{L}_{ij})}{({R}_{ij}+{L}_{ij})} \right|\right.$$

with R_ij_ and L_ij_ being the power spectral density of the signals for each electrode pairing (with i = 1, 2,…, M) at each frequency (with j = 1, 2,…, N). DAR was calculated as the sum of power of delta (1–3 Hz) frequencies divided by the sum of power of alpha (8–13 Hz) frequencies. We calculated DTABR as the sum of power of delta (1–3 Hz) and theta (4–7 Hz) frequencies divided by the sum of power of alpha (8–13 Hz) and beta (14–20 Hz) frequencies. In addition to these band-based measures of oscillatory power, we also analyzed the spectra using Fitting Oscillations & One-Over F (fooof)^30^, to determine the slope and intercept of the aperiodic component to see if these differed between groups. This analysis was performed using version 0.1.1 of the FOOOF MATLAB wrapper with the following settings: peak width limits = [0.5,12]; max number of peaks = Inf; minimum peak amplitude = 0.0; peak threshold = 2.0; background mode = fixed; verbose = true; frequency range = 0.5–30 Hz. Finally, using the on-board gyroscope and accelerometer, we calculated both the standard deviations and the root mean square (RMS) of the head movement over time to identify the differences in variability of movement across the X, Y, and Z movement planes.

Using data calculated in the previous analyses (Table 2), TreeBagger creates classification trees by utilizing the inputted data as base learners, then applying a bootstrapping algorithm to produce N number of trees (N = 200), then combines multiple trees to produce one final tree, thus reducing the effect of overfitting and improving generalization. We created three trees to predict: the severity of strokes, if strokes had occurred, and whether the participant fell into a more severe category (both moderate and large strokes combined).

**Table 2:** List of predictor variables used in making decision trees.

Predictor variables used in decision trees		
Demographic variables	EEG variables	Movement variables
Gender	pdBSI	Gyroscope RMS-X plane
Age	DAR-contralateral hemisphere	Gyroscope RMS-Y plane
	DTABR-contralateral hemisphere	Gyroscope RMS-Z plane
	Fooof background spectra intercept	Gyroscope standard deviation-X plane
	Fooof background spectra slope	Gyroscope standard deviation-Y plane
	Relative beta power	Gyroscope standard deviation-Z plane
	Relative alpha power	Accelerometer RMS-X plane
	Relative theta power	Accelerometer RMS-Y plane
	Relative delta power	Accelerometer RMS-Z plane
	High frequency pdBSI	Accelerometer standard deviation-X plane
	Low frequency pdBSI	Accelerometer standard deviation-Y plane
	PdBSI-frontal electrodes	Accelerometer standard deviation-Z plane

### Statistical analysis

All statistical analyses were completed in MATLAB with the significance threshold of α = 0.05. We used a one-way ANOVA to test for group differences in pdBSI. We ran 2-way ANOVA tests on the outcomes of the DAR, DTABR, gyroscope, and accelerometer data with factors of severity and hemisphere. Post-hoc group comparisons were done using the Tukey–Kramer correction. All data are presented as mean ± standard error of the mean (SEM). We calculated accuracy (ACC), sensitivity (SE) and specificity (SP) as a comparison of each classification tree output and the outcome of clinical diagnoses (Eqs. 2–4, respectively). These calculations used the number of true positives (TP), false positives (FP), false negatives (FN), and true negatives (TN)^31^. OOBerror was used to estimate these values.

2 $$ACC= \frac{TP+TN }{TP+TN+FP+FN}$$

3 $$SE= \frac{TP }{TP+FN}$$

4 $$SP= \frac{TN }{TP+FN}$$

## Results

**Figure 1:**
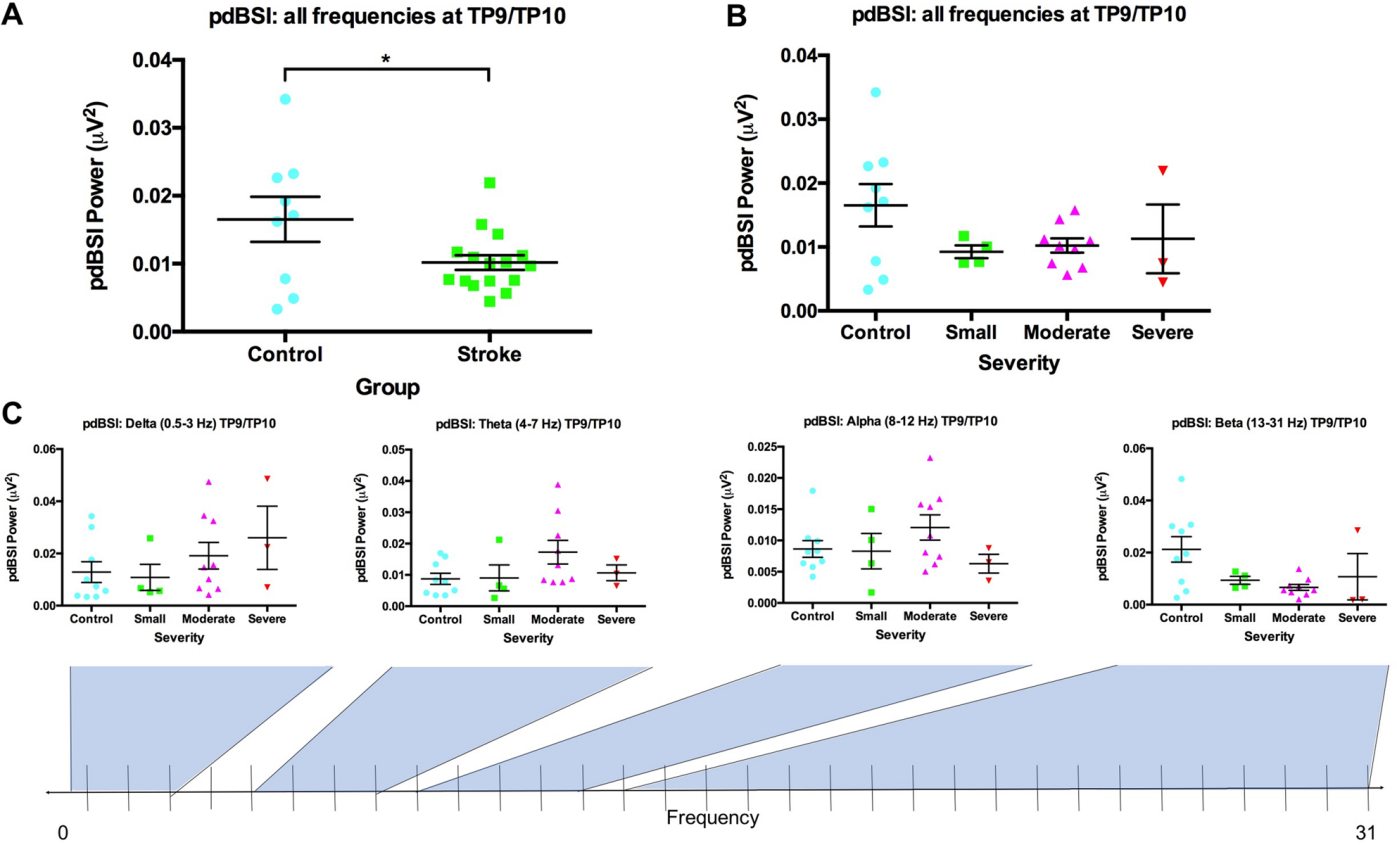
Pairwise Derived Brain Symmetry Index by group and frequency. Pairwise derived brain symmetry index by group across all frequencies (1–30 Hz) at electrodes TP9 and TP10 (**A**), pdBSI by severity across all frequencies (**B**), pdBSI by severity for each frequency bin (**C**). Standard error was calculated for each bar. *Significance at p < 0.050. Brain activity is more symmetrical after stroke. Average pdBSI does not readily distinguish between stroke severities. Data is presented as mean ± SEM.

**Figure 2:**
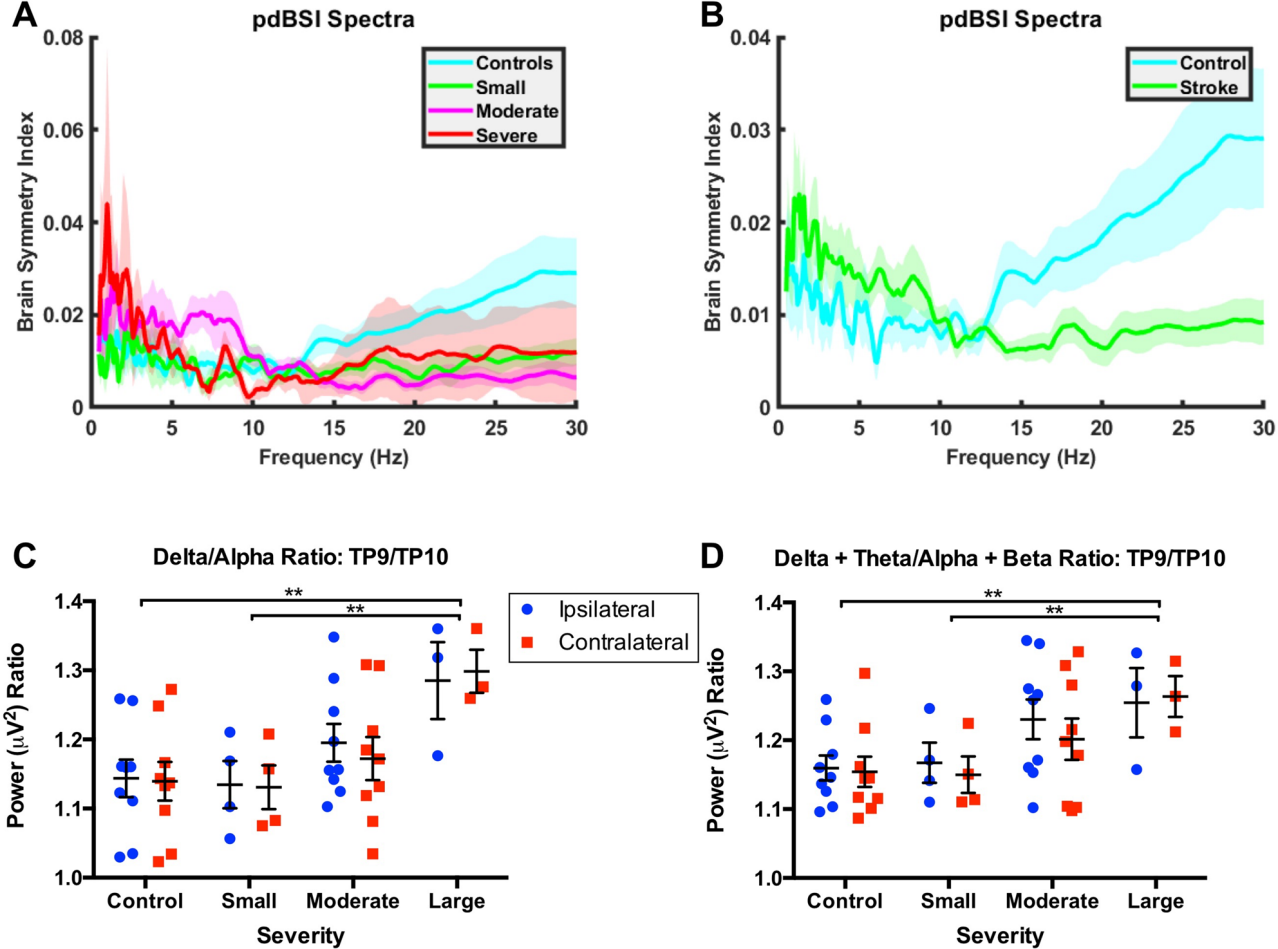
Pairwise Derived Brain Symmetry Index across frequency by stroke severity at electrodes TP9 and TP10, DAR across severities, and DTABR across severities. **Significance at p = 0.01. In stroke patients, pdBSI increases at low frequencies (**A**). However, above ~ 10 Hz, pdBSI decreases in stroke patients. When stroke patients are grouped by severity (**B**), it is difficult to differentiate severity by pdBSI at higher frequencies, largely due to high variability in the severe stroke group. DAR increases as stroke severity increases in moderate and severe strokes (**C**). We do not observe any clear hemispheric differences in DAR. DTABR increases with increasing severity (**D**). No clear hemispheric differences have been observed, but in small and moderate stroke there is a trend for increased slowing on the injured hemisphere. Data are presented as mean ± SEM.

The analysis of pdBSI showed significant differences between patients with strokes and the healthy controls (p = 0.036, F = 4.96, df = 1, 23, partial η^2^ = 0.177). Overall, brain activity was more symmetrical in stroke patients (Fig. 1A). Changes in pdBSI were dependent on the frequency range, as seen in Fig. 1C. In stroke patients, brain symmetry decreased at lower frequencies and increased at higher frequencies, whereas the opposite is true for the controls (Fig. 2). pdBSI also showed a trending severity effect, although insignificant as indicated by post-hoc tests (Fig. 1B). This severity effect was frequency-dependent and was driven by the beta frequency range, while an opposing effect is seen in the delta range as expected from the spectra in Fig. 2.

We calculated DAR as seen in Fig. 2C; we found that DAR increased with increasing stroke severity, particularly in the moderate and severe groups. There was a significant difference between DAR of those with large strokes and both controls and those in the small stroke category (p = 0.0021, F = 5.79, df = 3, 42, partial η^2^ = 0.293). We saw similar results when we calculated the DTABR (p = 0.01, F = 4.29, df = 3, 42, partial η^2^ = 0.234), as seen in Fig. 2D. In both DAR and DTABR, there was an increased power in the ipsilateral side of the brain for patients in the large stroke category. This effect was insignificant; however, an interesting effect to note as the other severities and controls showed the opposite effect.

**Figure 3:**
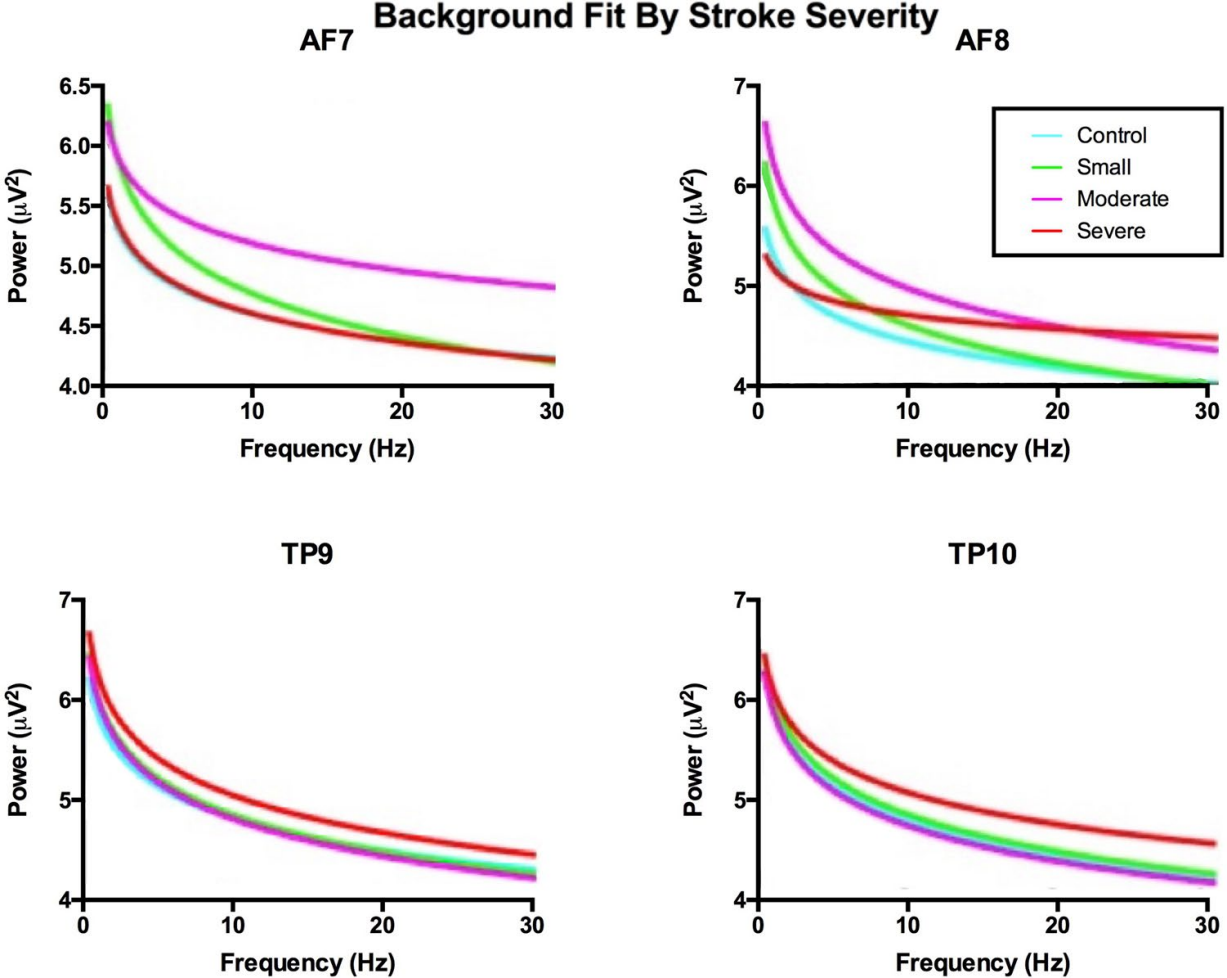
Background fit of data from fooof analysis at each electrode by severity. Non-oscillatory activity was not different between severities, indicating that band-ratios are not affected by background brain activity levels.

We analyzed the general spectra using fooof to determine if the band ratio effects were due to non-oscillatory changes in brain activity (Fig. 3). Specifically, the slope of the background spectra did not change between severities (p = 0.6336, F = 0.58, df = 3, 21). The intercepts of the of the background spectra also had no changes between severities (p = 0.7743, F = 0.37, df = 3, 21).

**Figure 4:**
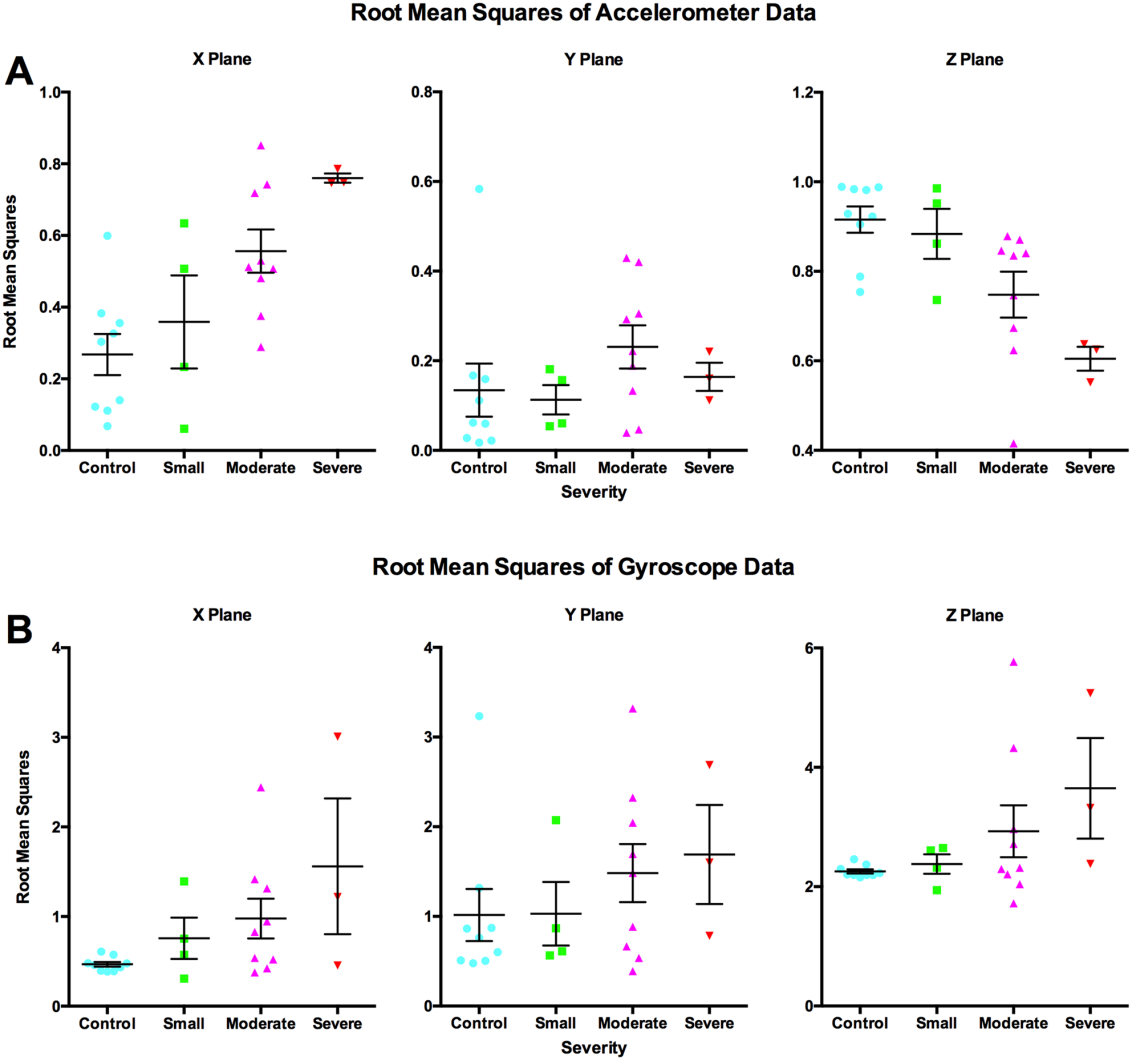
RMS for accelerometer (**A**) and gyroscope (**B**) across three planes of movement. RMS of accelerometer data increased with increasing stroke severity in the X-plane and decreased with stroke severity in the Z-plane (**A**). No significant group differences in the gyroscope data (**B**). Data are presented as mean ± SEM.

There was a significant interaction between severity and axis of movement in the RMS accelerometer data (p ≈ 0, F = 7.13, df = 6, 63 partial η^2^ = 0.404). Qualitative analysis demonstrates that this interaction effect showed no differences in the Y-axis and increased with severity in the Z-axis, while decreasing with severity in the X-axis (Fig. 4). Data from the gyroscope had no significant differences; however, the variability in movement is more consistent among controls when compared to the other groups, in all planes.

**Figure 5:**
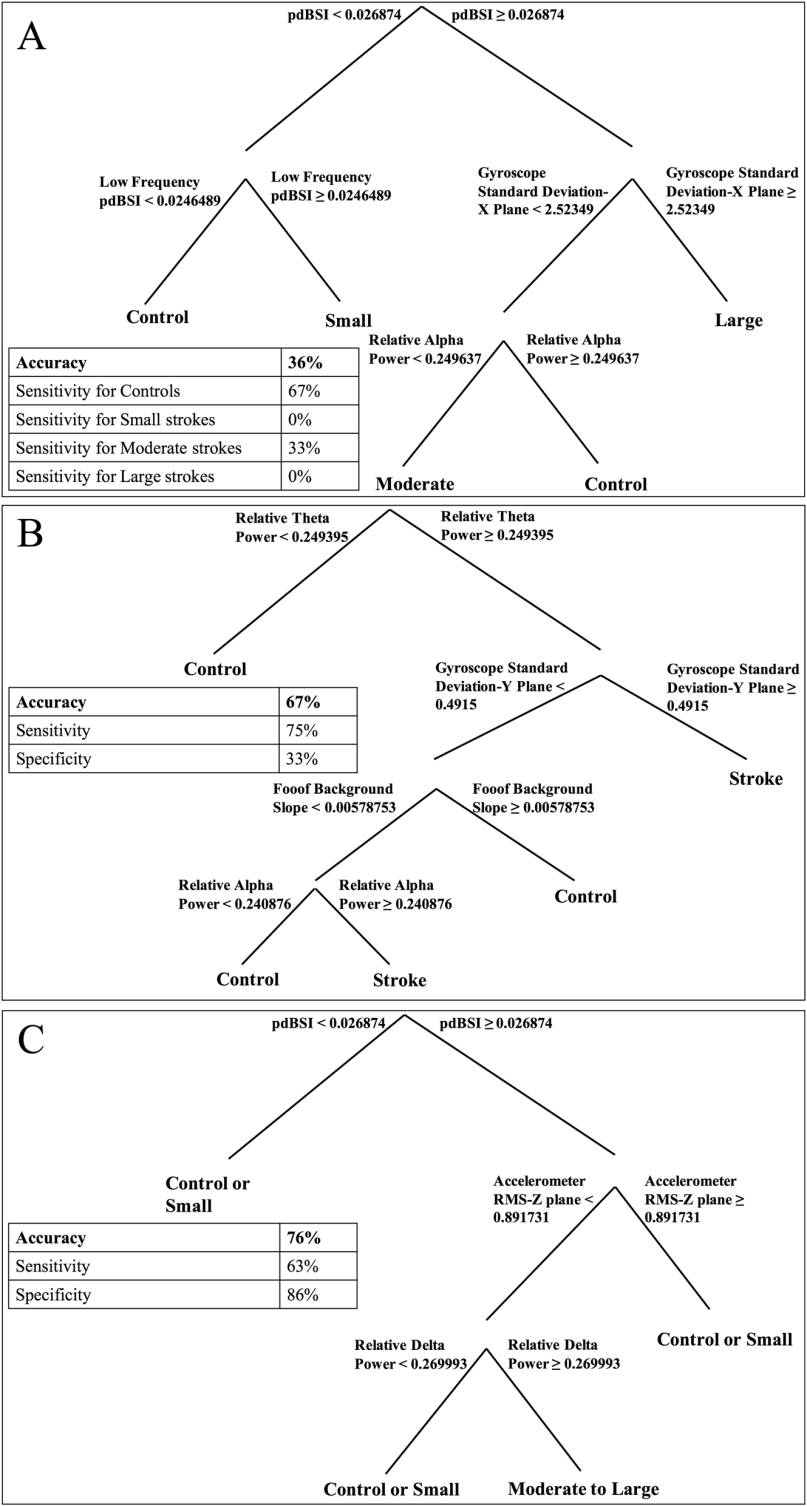
Classification trees for determining (**A**) stroke severity, (**B**) presence of stroke, (**C**) higher-risk stroke. Predicting stroke severity was the least accurate model and predicting more severe strokes was the most accurate model.

The severity classification tree has an accuracy of 0.36 and sensitivity for controls, small strokes, moderate strokes, and large strokes, of 0.67, 0, 0.33, and 0, respectively, as seen in Fig. 5A. The classification tree that groups by stroke or control has an accuracy of 0.67, with a sensitivity and specificity of 0.75 and 0.33, respectively (Fig. 5B). The final classification tree groups by moderate and large strokes or small strokes and controls has an accuracy of 0.76, with a sensitivity and specificity of 0.63 and 0.86, respectively (Fig. 5C).

## Discussion

These results indicate that stroke and stroke severity can be detected with the Muse EEG system. Brain symmetry differs between stroke patients and controls at certain frequencies. Additionally, DAR and DTABR are increased in moderate and severe strokes, indicating that there is a slowing in brain activity. Further, the Muse set up took approximately 5 min and was tolerated even by patients with more severe deficits, making this system feasible for future use in an ambulance. The qEEG measures used, including pdBSI and measures of slowing, can be quickly calculated in a prehospital setting, and interpretation could further be automated, such as in an application that could both process and analyze EEG samples from a suspected stroke patient. The short set-up time, combined with simple qEEG measures, makes this system a promising method for distinguishing strokes from stroke mimics and identifying those strokes associated with LVO that need to be triaged preferentially to comprehensive stroke centres with endovascular thrombectomy capability.

Our findings contradict the findings of many papers in regards to increased asymmetry after stroke^7,10,32^. Many studies that assess brain symmetry compare average power, rather than power across frequencies, as we have done here. This alternative method may mask differences in pdBSI across frequencies, such as our observed increased asymmetry at lower frequencies and decreased asymmetry at higher frequencies. Further, many researchers compute pdBSI from 1 to 25 Hz, whereas we found the most pronounced differences in pdBSI in frequencies greater than 20 Hz, which may be driving the differences seen. The differences in the 20–30 Hz frequencies likely are, in part, caused by muscle artifacts, which can occur between 20 and 300 Hz. Regardless, these measures show promise in being indicative of stroke severity. A recent pilot study found, using the Muse, that stroke patients had increased brain asymmetry compared to controls using the revised brain symmetry index^27,33^. This is contrary to our findings, which show overall decreased asymmetry in the stroke patients. The revised BSI compares the symmetry at each frequency across the electrodes, whereas the pdBSI considers the symmetry between each homologous pair of electrodes at each frequency^10^. In this case, we compared symmetry using one homologous pair of electrodes, so this would not affect these results, but may affect comparisons between other studies of brain symmetry after stroke. Gottlibe et al. filtered their data from 0.16 to 76 Hz and therefore may be picking up on muscle artifacts in the higher frequency range. When we analyzed our existing data to include the 0.16–76 Hz, we still see overall increases in brain symmetry, contradicting previous findings. This could be due to differences in the way studies were conducted (e.g., patients sitting vs lying down), which would lead to differences in the way muscle artifacts affect the data^34^. In this study, we show that reducing brain symmetry measures to a single number may be masking important information, such as the symmetry differences between lower and higher frequencies.

Here, we found an increase in DAR and DTABR in moderate and severe strokes. This increase indicates a slowing in brain activity due to ischemia and likely impaired CBF, similar to the findings of other studies in acute stroke^9,35^. Interestingly, we did not see a significant increase in either DAR or DTABR in small strokes, indicating that this measure may be useful in distinguishing strokes by severity and identify those patients most likely to have LVO. This effect of stroke severity on DAR is supported by studies that show DAR predicts outcome and relates to stroke recovery^10,18^.

With the Muse, we can also look at participant movement using data obtained from the accelerometer and the gyroscope during recording. Here, we found that stroke patients had more variability in acceleration in the X-axis and less in the Z-axis, which increased with increasing severity. This finding equates to more movement in tilting the head from left to right (as in, tilting ear to shoulder), and less movement shaking the head from left to right (as in, “shaking your head no”). These findings may indicate movement differences due to motor system impairments or attentional differences due to contralateral neglect. However, since data was collected with eyes closed, we believe differences are likely due to impaired motor function, including postural support. Interestingly, there were no differences in the movement data from the gyroscope, which can also measure rotation. This indicates that the accelerometer may be a more useful tool for detecting movement differences between stroke patients and controls, and that differences in general movement may be more predictive than rotational differences.

In the literature, there are multiple qualitative and quantitative aspects of EEG that may differentiate stroke patients from controls. Here, we used decision trees to determine which of the quantitative EEG and movement measures we looked at were the most useful in determining which participants had a stroke and to which severity category they belonged. We used the TreeBagger function in MATLAB, which controls for overfitting to the sample population by creating bootstrap-aggregated decision trees. Thus, although we have “wide data,” with many predictor variables and a small sample size, this method mitigates some of the risks of overfitting. Here, we found that our model for determining severity had low accuracy and had difficulty (0% sensitivity) at classifying small strokes and large strokes (Table 3). However, model accuracy improved when data was used to determine if participants were in the stroke group (any size) or control group. This model had 67% accuracy, 75% sensitivity, and 33% specificity. However, the accuracy of this model was not much higher than a baseline classifier (64%). Features included in this tree were: relative theta and alpha power, variability in the Y-axis using the gyroscope, and the slope of the background spectra (the aperiodic component of the EEG spectra). The model improved further when classifying the more severe moderate and large strokes from minor stroke and control patients. This classification is of clinical relevance since it approximates the prediction that paramedics make when deciding whether suspected strokes require transport to a specialized stroke centre vs. a standard hospital. This model had 76% accuracy, 63% sensitivity, and 86% specificity. This was the most accurate model when compared to the chance level of prediction (52%, Table 3). For comparison, currently used prehospital stroke scales such as the Los Angeles Motor Scales which are designed to designed to detect stroke from large vessel occlusion have reported a comparable accuracy of 0.72^36^. Future studies are needed to determine the additive value of the Muse headband. Important predictors in this model were: brain symmetry, variability in the Z-axis using the accelerometer, and relative delta power. These decision trees provide a simple way to predict stroke incidence and stroke severity using multiple measures from the Muse instead of relying on one index (Table 3).

**Table 3:** Confusion matrix values for each model as well as the chance rate of a baseline classifier.

Model	True negative	False negative	True positive	False positive	Chance accuracy (%)
Control vs. small vs. moderate vs. large	Control: 8 Small: 19 Moderate: 13 Large: 20	Control: 3 Small: 4 Moderate: 6 Large: 3	Control: 6 Small: 0 Moderate: 3 Large: 0	Control: 8 Small: 2 Moderate: 3 Large: 2	36
Control or minor stroke vs. moderate-large	12	4	7	2	52
Control vs. stroke	3	4	12	6	64

The first model had four groups, and thus the true negative, false negative, true positive, and false positive rate for each subgroup was compared to the combination of the other groups to allow for calculation.

Our study has a few notable limitations. First, we assessed patients an average of 3.71 days (range: 0–16 days) after stroke onset. In the subacute phase of stroke, as we have assessed in this study, reperfusion and restoration of CBF likely change the EEG signatures as compared to the hyper-acute phase of stroke, when CBF is severely decreased. Indeed, different levels of CBF are associated with different EEG frequency alterations^8^. However, assessing patients in the subacute phase was more practical for this study as the goal was to establish the initial feasibility of this stroke detection method. Second, we have a small sample size and are likely underpowered to detect effects. When analyzing the data by stroke severity, hemisphere, location of stroke, etc., the subgroups have a small sample size that may not be representative of that population. Therefore, our follow up study will validate these findings using a much larger sample size so that subgroup analyses will be adequately powered. Third, scalp measures of EEG measure cortical activity, and the effects of stroke on cortical activity likely vary depending on stroke location^12^. In this sample, only three stroke patients had subcortical stroke, leaving us underpowered to detect effects of stroke location on EEG. However, when using classification trees to predict stroke location, none of the EEG measures significantly predicted stroke location (model R^2^ = 0.000171), suggesting that stroke location had minor effects on EEG measures and that EEG disturbances caused by stroke may be largely global. Finally, prehospital stroke scale scores were not available for study patients. As a result, it remains unclear to what extent EEG will improve the diagnostic accuracy above that of currently used EMS stroke scales.

In summary, our results suggest that qEEG measures, such as pdBSI, DAR, DTABR, and head movement variability can distinguish stroke severities when measured using a low-cost bedside EEG recording. These findings warrant further study in a large sample and at an earlier time after stroke onset, as brain activity changes with the progression of stroke injury. Future work will assess patients earlier, such as in the emergency department or in the ambulance, to determine the utility of using EEG in the hyper-acute phase of stroke. Additional studies will help further elucidate the feasibility and utility of using portable EEG as an inexpensive and early detection tool to use in ambulances to aid in the diagnosis of stroke and the detection of stroke from LVO.

## Data Availability

Anonymized data will be shared upon request from qualified investigators.

## Code availability

https://github.com/APPLabUofA/Muse_Stroke_Study/blob/master/README.md.
